# Physiological pathways linking body conformation and reproductive efficiency in Murciano-Granadina does: A novel regularized canonical correlation perspective

**DOI:** 10.1016/j.vas.2026.100632

**Published:** 2026-03-24

**Authors:** María Pía Peláez Caro, Ander Arando Arbulu, José Manuel León Jurado, Juan Vicente Delgado Bermejo, Javier Fernández Álvarez, Francisco Javier Navas González

**Affiliations:** a Department of Genetics, Faculty of Veterinary Medicine, University of Córdoba, Córdoba, Spain; b National Association of Breeders of Murciano-Granadina Goat Breed, Granada, Spain; c Centro Agropecuario Provincial de la Diputación de Córdoba, Córdoba, Spain

**Keywords:** Artificial insemination, Udder conformation, Skeletal robustness, Dairy goat breeding, Fertility resilience

## Abstract

This study examines the relationship between linear appraisal and long-term fertility dynamics in Murciano-Granadina goats, analyzing a dataset of 21,757 does and 32,693 artificial insemination (AI) records collected over 10 years. Fertility was assessed by day of insemination, buck batch, and semen type (fresh/chilled vs. frozen/thawed). Descriptive fertility rates revealed mean values of 52.6 % (day of insemination), 53.4 % (buck batch/day), and 48.3 % (semen type). Canonical correlation analysis (rCCA) showed significant but moderate associations between fertility and morphology (canonical correlations: 0.191, 0.144, and 0.045). First two canonical functions explained 96.7 % of variability, though redundancy coefficients were low (≤0.0146), indicating that morphology accounted for only a moderate proportion of fertility variability. Specific LAS traits provided subtle insights: chest width and rump width negatively correlated with fertility (*r* = –0.14 and –0.11, respectively), while bone quality showed the highest positive association (*r* ≈ 0.05). Rear udder insertion height and hind leg side view were positively linked to fertility resilience under frozen/thawed semen use. Conversely, excessively deep udders and large body size tended to compromise fertility, likely through mastitis risk or negative energy balance. Findings suggest skeletal robustness, udder attachment, and hind leg conformation support both fertility and productive longevity, while extreme dairy specialization may reduce reproductive outcomes. Overall, fertility in Murciano-Granadina does is multifactorial and moderately explained by linear appraisal traits, hence integrating morphological evaluation with artificial insemination strategies may enhance selection decisions. Future breeding programs should prioritize bone quality, functional udder morphology, and locomotor soundness to balance milk productivity with reproductive efficiency.

## Highlights

• AI with linear appraisal enhances fertility management in dairy goats.
• Skeletal robustness and hind leg soundness improve fertility resilience.
• Angular frames and compact udders show links with fertility variation.
• Bone quality and udder attachment emerge as key reproductive traits.
• Excessive stature and deep udders reduce reproductive efficiency.

## Graphical abstract

**Figure:**
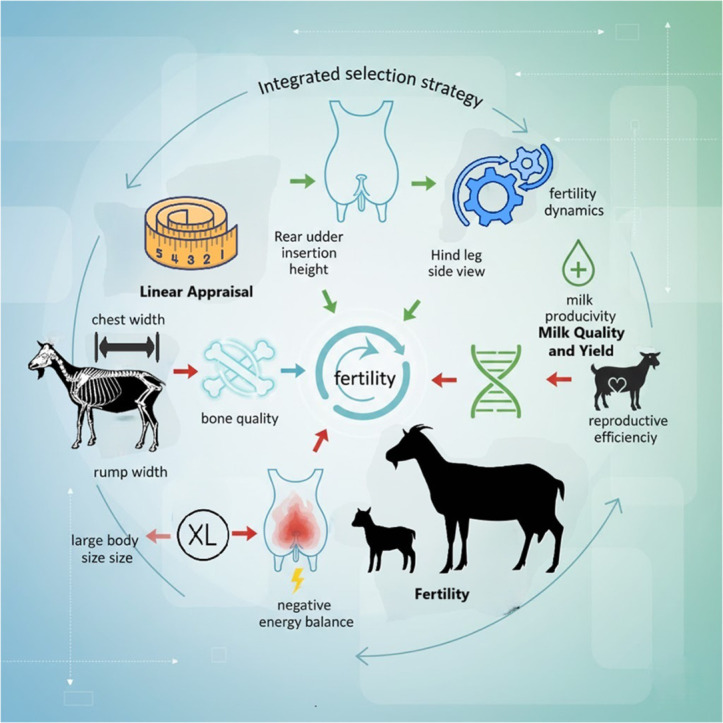

## Introduction

The Murciano-Granadina goat breed is one of Spain’s most significant and widely distributed breeds, celebrated for its large population, robust selection practices, and broad geographic reach. Over the last decade, the breed has seen remarkable growth, both in Spain and internationally. This expansion is primarily due to the breed’s high rusticity, adaptability to various climates, and its suitability for diverse production systems, making it increasingly popular worldwide (National Association of Breeders of Murciano-Granadina Goat CAPRIGRAN, 2021; Delgado et al., 2017; León et al., 2012).

The National Association of Breeders of the Murciano-Granadina Goat Breed (CAPRIGRAN) has played a pivotal role in the breed’s genetic improvement. Through its breeding program, CAPRIGRAN has focused on not only enhancing productivity but also on selecting animals with high-quality milk and long lifespans. The breeding program integrates modern tools like productive control, which is based on official milk yield data, and the linear appraisal system (LAS), an evaluation method that assesses 17 key zoometric traits on a 1–9 scale. These traits have demonstrated strong genetic and phenotypic links to important factors such as growth, reproduction, longevity, and milk production (Fernández Álvarez et al., 2020; García-Ballesteros et al., 2017; Bukar-Kolo et al., 2016). This appraisal system is central to the goat qualification process, providing a standardized measure of key traits that directly impact the breed’s success.

One of the key advancements in the breed's genetic improvement is the use of artificial insemination (AI). AI allows for the genetic connection of herds, enabling breeders to spread desirable traits without compromising the animals' health. It facilitates the transmission of superior genetics, which enhances productive and morphological traits across herds while reducing the risks associated with natural breeding (Arando Arbulu et al., 2021; Arrebola et al., 2013; Cseh et al., 2012). This technology also plays a significant role in advancing genetic progress, especially in optimizing the breed’s productivity and reproductive outcomes.

However, while the linear appraisal system has made clear connections between morphological traits, such as udder development and milk production, the relationship between these traits and fertility is less well understood. The complex nature of fertility, particularly in the context of artificial insemination, complicates the identification of direct correlations. Despite this challenge, understanding how zoometric traits influence fertility could improve breeding programs and lead to more efficient management of resources, reducing costs associated with lost lactations, transportation, and the labor of technicians involved in insemination (Nunes & Salgueiro, 2011; Tadesse et al., 2022).

Morphological traits are known to serve as reliable indicators of reproductive capacity and milk production potential. Studies in both goats and other livestock species have explored how certain physical characteristics influence reproduction. For instance, research has shown that udder and leg structure are correlated with fertility in goats, with well-formed fore udders and healthy median ligaments associated with reduced risks of stillbirths (Mellado et al., 2008; Peris et al., 1999; Samad, 2022). Other studies, such as those by Mellado et al. (2008), have reinforced this, highlighting how morphology can impact birth outcomes. Furthermore, testicular traits in males have been linked to better breeding success, with larger, well-developed testes correlating with higher sperm production and improved fertility rates (Mekasha et al., 2008).

While much of this research has focused on specific traits, the relationship between larger body size and reproductive success is also significant. In goats, larger animals, particularly those with superior udder development, tend to have more favorable fertility outcomes. This connection suggests that body size and morphology are intertwined with the breed’s reproductive potential (Zuñiga-Garcia et al., 2020).

Understanding how zoometric traits affect fertility in the Murciano-Granadina breed can offer crucial insights for optimizing breeding programs aimed at enhancing both productivity and reproductive success. By identifying traits that positively influence reproductive outcomes, breeders can refine their selection criteria, leading to more efficient and effective breeding strategies. This, in turn, will contribute to the breed’s ongoing success and help maintain its growing popularity (Assan, 2020).

The aim of this paper is to investigate the regularized canonical correlations between linear appraisal traits and reproductive success in Murciano-Granadina does. Specifically, this study seeks to identify which zoometric traits, assessed through linear appraisal, are most closely associated with fertility outcomes. By exploring the pathways that connect these traits to reproductive success, the paper aims to provide insights that can inform breeding strategies designed to enhance both the reproductive efficiency and overall productivity of the breed.

Body size and overall condition are among the most direct factors affecting fertility. Goats with optimal body condition generally experience better reproductive efficiency due to higher energy reserves, which are essential for reproduction. Larger goats with adequate body mass tend to have better ovulation rates and more successful breeding outcomes. In contrast, underweight or poorly developed animals may face delayed puberty or anovulation, while excessively large goats may experience birthing difficulties or reduced reproductive health (Bukar-Kolo et al., 2016).

The structure of the udder and mammary system is similarly important for both milk production and reproductive success. A well-developed udder, with proper fore udder attachment and healthy median ligaments, is crucial for successful lactation post-kidding. These traits not only reflect an animal's general health but also play a role in fertility. Poorly attached udders or overly refined udders can result in low milk yields and higher mastitis rates, which in turn can affect hormonal balance and reduce the goat's ability to conceive again after giving birth (Fernandez Alvarez et al., 2023; Peris et al., 1999; Setiati et al., 2023).

Pelvic structure and the alignment of the rear legs also influence fertility by ensuring smooth labor. A well-structured pelvic cavity facilitates easier births, which is vital for the health of both the mother and offspring. Narrow pelvises or poorly aligned legs can lead to dystocia, or difficult births, which often require veterinary intervention and can reduce breeding success (Mocé et al., 2022). Thus, pelvic width and rear leg conformation are key traits that impact fertility.

For male goats, the size and development of the testes are crucial indicators of reproductive success. Larger, well-developed testes produce higher volumes of sperm, improving the chances of successful breeding. Early testicular development is a reliable predictor of future reproductive performance, making this trait particularly important when selecting breeding males (Mekasha et al., 2008; Bukar-Kolo et al., 2016).

The leg structure and mobility of goats also play a role in fertility. Goats with good leg conformation are more likely to be active during estrus, which facilitates successful mating. Conversely, poor leg structure can limit mobility and hinder mating behavior, leading to reduced chances of conception (Peris et al., 1999).

The development of reproductive organs is directly correlated with larger body size and better-developed udders, both of which signal an animal's overall health and reproductive capacity. Adequate energy reserves, derived from fat and muscle mass, support reproductive success by ensuring the goat can carry and nourish offspring. Goats with good body condition are more likely to experience regular estrus cycles and achieve higher conception rates (Tadesse et al., 2022; Bukar-Kolo et al., 2016).

Many of the traits assessed in linear appraisal systems are genetically controlled, which means that desirable traits like udder structure, pelvic width, and body size can be passed down to future generations. Identifying traits linked to fertility allows for targeted genetic selection, which can improve the fertility rates and productivity of herds over time (García-Ballesteros et al., 2017; Fernández Álvarez et al., 2020).

By selecting goats based on traits that correlate with fertility, breeders can optimize breeding programs to improve reproductive outcomes and herd productivity. For example, goats with larger pelvic structures, better udder attachments, and more symmetrical leg alignment are more likely to experience successful breeding, ultimately leading to higher fertility rates and better milk production. These traits should be prioritized in selection criteria, ensuring that future generations exhibit improved reproductive efficiency (Arando Arbulu et al., 2021; Assan, 2020).

The integration of artificial insemination (AI) further enhances the potential for genetic progress. By using semen from males with superior reproductive traits, breeders can spread these traits across herds, improving both fertility and overall productivity. AI also allows for pairing genetically superior females with top-quality sires, optimizing fertility outcomes (Arrebola et al., 2013; Peláez Caro et al., 2024).

Understanding the relationship between zoometric traits and fertility enables better management practices, including improved mating schedules, nutritional strategies, and health monitoring. Goats in optimal condition tend to exhibit more predictable estrus cycles, making it easier to breed them and increase conception rates. Fewer reproductive failures lead to more efficient herd management, ultimately reducing costs associated with veterinary interventions, transportation, and labor (Nunes & Salgueiro, 2011; Tadesse et al., 2022).

The aim of this paper is to explore the relationship between linear appraisal traits and reproductive success in Murciano-Granadina female goats (does). Specifically, the study focuses on identifying key zoometric traits that influence fertility outcomes. By examining how various morphological traits—such as body size, udder development, pelvic structure, and leg conformation—correlate with reproductive success, the paper aims to provide insights into optimizing breeding programs. The overall goal is to enhance both productivity and reproductive efficiency in the Murciano-Granadina breed, offering a better understanding of how selecting for specific traits can improve fertility and inform herd management strategies.

## Material and methods

### Sample and study conditions

**Fig. 1:**
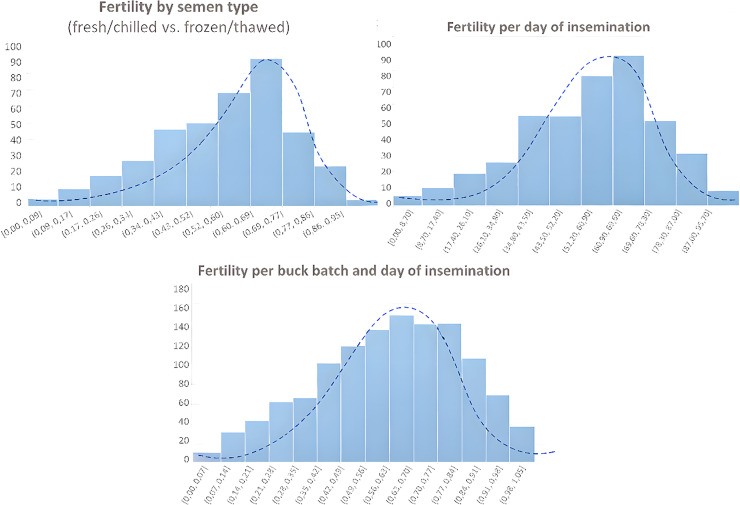
Frequency distributions of fertility percentages according to semen type (fresh/chilled vs. frozen/thawed), day of insemination, and buck batch combined with insemination day. Histograms represent empirical fertility frequencies, with superimposed kernel density estimations and fitted Gaussian curves illustrating overall distributional patterns. The predominantly unimodal and approximately symmetric shapes suggest fertility values approaching normality, thereby supporting the classification of fertility categories based on central tendencies and empirical distribution tails.

**Fig. 2:**
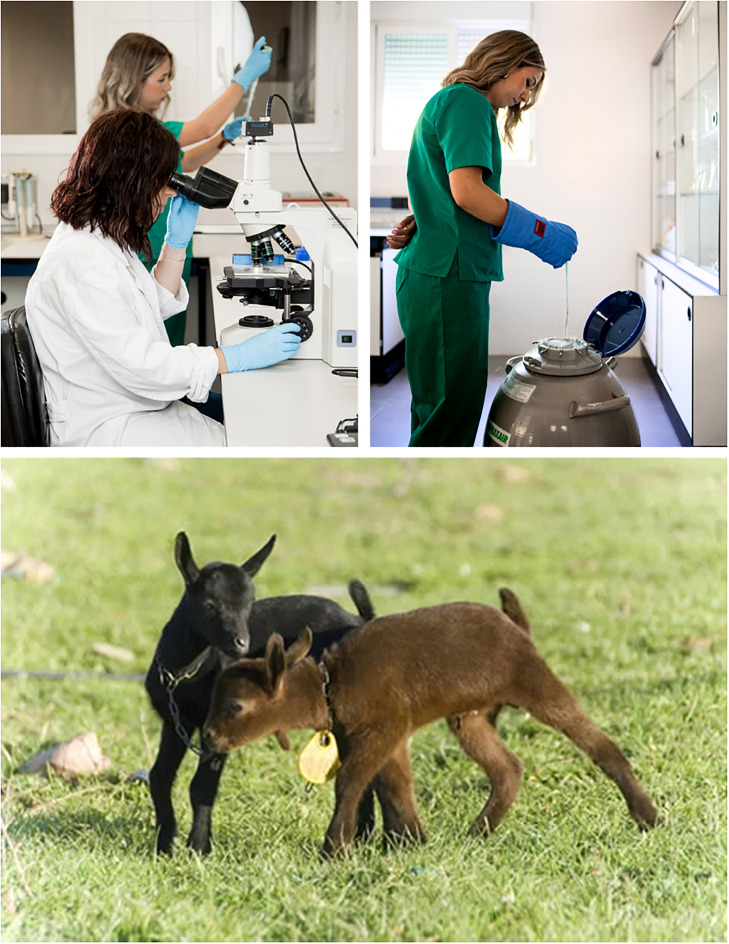
Caprigén (Andalusian Goat Selection and Improvement Center) Semen sampling Process (top left and right) and Murciano-Granadina kids (bottom).

This longitudinal study spanned a 10-year period (2010–2019) and included 21,757 Murciano-Granadina does born between September 1999 and June 2018. Over this time, a total of 32,693 artificial insemination (AI) records were collected from January 2010 to December 2019. Animals were not selectively recruited to constitute the study sample. All does for which both linear appraisal (LAS) records and corresponding fertility data were available during the study period were included in the analysis, ensuring that the dataset represents the complete recorded breeding population meeting these objective data availability criteria rather than a subgroup selected for reproductive performance. These animals belong to the official Murciano-Granadina breeding program, in which reproductive efficiency forms part of the structured selection objectives. Thus, although no additional fertility-based filtering was applied for research purposes, the population reflects ongoing genetic improvement for productive and functional traits. The empirical distribution of the fertility indicators across semen type, insemination day, and buck batch is illustrated in Fig. 1. Semen was obtained from 115 Murciano-Granadina bucks enrolled in the national breeding program, all of which had been selected for proven fertility and high genetic merit (Fig. 2).

### Doe location

**Fig. 3:**
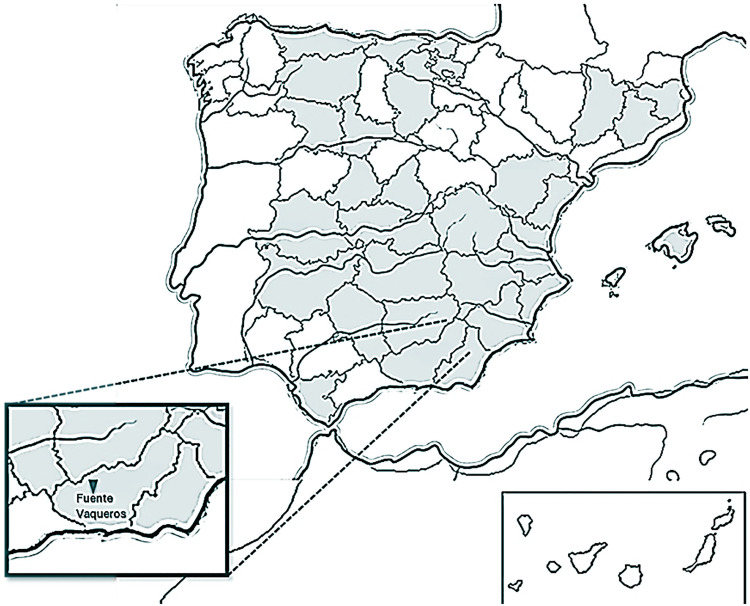
Murciano-Granadina goat breed distribution across Spain and location of Caprigén (Andalusian Goat Selection and Improvement Center) in Fuente Vaqueros (66GP+6X Santa Fe, Granada, Spain).

**Fig. 4:**
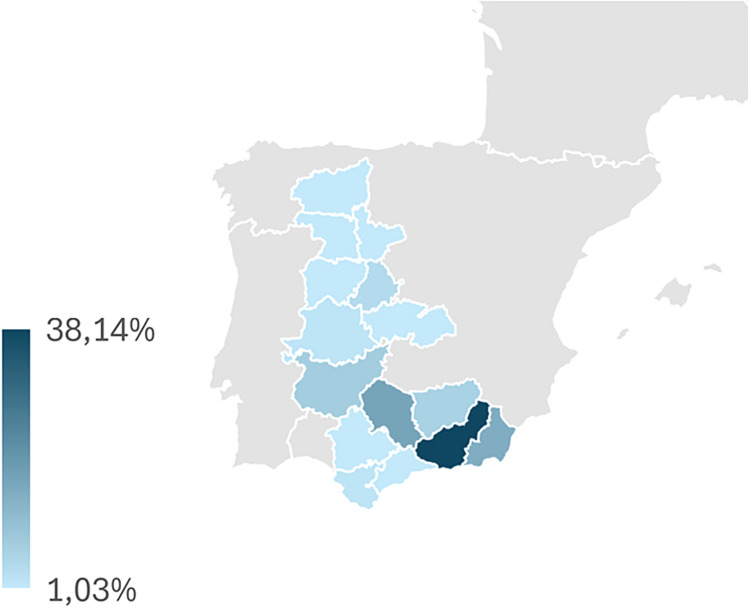
Geographic distribution of the Murciano-Granadina goat farms included in the study across 16 provinces in Spain and Portugal. The darkest shading corresponds to Granada, which concentrated the highest proportion of farms (38.1 %), while other provinces represented smaller shares (down to 1.0 %). This highlights the historical and current importance of southeastern Spain as the main nucleus of Murciano-Granadina goat breeding.

Fig. 3 illustrates the distribution of the Murciano-Granadina breed across Spain. Supplementary Table S1 provides detailed geographic information for participating farms, including farm acronym, country, province, town, and precise latitude and longitude and Fig. 4 shows the concentration of farms participating in the study across the Spanish territory. These geolocations are critical for evaluating environmental and logistical factors affecting reproductive performance and for enabling accurate spatial mapping in management and ecological studies.

### Buck location

Bucks were housed at the Andalusian Goat Selection and Improvement Center in Albolote (68HQ+3R, Granada, Spain) until 2014, after which they were relocated to Fuente Vaqueros (66GP+6X, Santa Fe, Granada, Spain). Both facilities are situated in the Vega de Granada, a continental Mediterranean region characterized by marked diurnal temperature variations often exceeding 20 °C, concentrated rainfall from late autumn to early spring, frequent frost events (6.2–8.7 days per season), and a mean annual precipitation of ∼419.5 mm. Throughout the study, bucks were managed under standardized housing and feeding conditions, receiving 0.5 kg of commercial concentrate daily, ad libitum hay and water, and mineral supplementation to ensure dietary consistency.

## Semen collection and preparation

Ejaculates were collected with an artificial vagina and immediately placed in a 37 °C water bath for evaluation. Only samples with volume > 0.5 mL, sperm concentration > 3000 × 10^6/mL, and mass motility > 4 were used for insemination.

• Chilled semen: Extended with Inra 96 (IMV Technologies, France) to 200 × 10^6 sperm per straw, stored at 5 °C, and used within 4–6 h.
• Cryopreserved semen: Extended with Triladyl (IMV Technologies, France) to 150 × 10^6 sperm per straw, frozen using a Digitcool programmable cryo-freezer, and thawed at 37 °C for 30 s prior to use in the field.

## Artificial insemination and pregnancy diagnosis

AI was carried out on 110 commercial Murciano-Granadina farms, approximately 46 h after sponge removal, using cervical insemination with a speculum and integrated illumination. Pregnancy was diagnosed 42 days post-insemination by transabdominal ultrasonography (5 MHz probe), and kidding records were used to confirm progeny origin.

## Fertility evaluation

Fertility was evaluated through three indicators:

1. Fertility per day of insemination,
2. Fertility per buck batch and day of insemination,
3. Fertility by semen type (fresh/chilled vs. frozen/thawed).

For canonical correlation analysis, fertility percentages were classified into five standardized categories: Very Low (≤ 20 %), Low (20–40 %), Medium (40–60 %), High (60–80 %), and Very High (80–100 %). This ensured consistent assessment of the effects of insemination timing, buck batch, and semen preservation method.

## Murciano-Granadina linear appraisal system (LAS)

The Murciano-Granadina LAS provides standardized morphological evaluations. Scores are assigned by trained raters in four categories for primiparous and multiparous does: (i) structure and capacity, (ii) dairy structure, (iii) mammary system, and (iv) legs and aplomb. For bucks, young males, and nulliparous females, only three categories are assessed, excluding the mammary system.

• Traits scored: 17 linear traits (primiparous/multiparous does) or 10 traits (bucks and young males) on a 9-point scale. Some traits (e.g., body depth, dairy structure, legs and aplomb) are assessed identically across sexes.
• Weighting of final score: For lactating does, 25% structure and capacity, 15% dairy structure, 20% legs and aplomb, and 40% mammary system. For bucks/young males, 50% structure and capacity, 20% dairy structure, 30% legs and aplomb.
  ○ Categorical ratings (CAPRIGRAN):
  ○ Insufficient (IN): ≤ 69 points (< 69%)
  ○ Mediocre (R): 70–74 points (70–74%)
  ○ Good (B): 75–79 points (75–79%)
  ○ Quite Good (BB): 80–84 points (80–84%)
  ○ Very Good (MB): 85–89 points (85–89%)
  ○ Excellent (E): > 90 points (≥ 90%)

Details of the scoring scales and conversion from zoometric measurements are provided in Table 1.

**Table 1:** Detailed description of the scales used and the translation process from zoometric traits to LAS scores in Murciano-Granadina primiparous and multiparous does.

Category	Trait	Range / Scale	Reference (LAS 5)	Optimum (Score)
Structure & Capacity	Stature (Height at Withers)	62–78 cm	70 cm	72 cm (Primipara, LAS 6); 74 cm (Multipara, LAS 7)
	Chest Width	15–23 cm	19 cm	20 cm (Primipara, LAS 6); 21 cm (Multipara, LAS 7)
	Body Depth	Shallow – Extremely deep	Intermediate (rib ≈ elbow end)	Deeper (LAS 7, both)
	Rump Width	13–21 cm	17 cm	18 cm (Primipara, LAS 6); 19 cm (Multipara, LAS 7)
	Rump Angle	55° (steep) – 31° (flat)	43°	31° (LAS 9)
Dairy Structure	Angulosity	Rough – Angulous	Intermediate	Angulous (LAS 9)
	Bone Quality	Round/rough – Flat/neat	Intermediate	Flat & neat (LAS 9)
Mammary System	Anterior Insertion	Weak – Strong	90°	120° (LAS 9)
	Rear Insertion Height	11–3 cm	7 cm	3 cm (LAS 9)
	Median Suspensory Ligament	1–9 cm	5 cm	5 cm (LAS 5)
	Udder Width	3–11 cm	7 cm	11 cm (LAS 9)
	Udder Depth	−10 (pendulous) → +10 cm (above hock)	0 cm (hock level)	−5 cm (Primipara, LAS 3); 0 cm (Multipara, LAS 5)
	Nipple Placement	90° (lateral) – 0° (vertical)	45°	0° (LAS 9)
	Nipple Diameter	0.5–4.5 cm	2.5 cm	2 cm (LAS 4)
Legs & Aplomb	Rear Legs – Rear View	Very close – Parallel/separated	Slightly close	Parallel/separated (LAS 9)
	Rear Legs – Side View	Straight – Very curved	Desirable curvature	Desirable curvature (LAS 5)
	Mobility	Poor stride – Long, strong, uniform stride	Moderate stride	Good mobility (LAS 9)

Age and lactation order were also recorded, as these factors influence LAS traits (Manfredi et al., 2001). In this dataset, lactation order correlated strongly with age (*r* = 0.705, *P* < 0.01). To avoid redundancy, lactation order was used as the main age-related variable, and analyses were stratified between primiparous and multiparous does (Wiggans & Hubbard, 2001).

Table 2.

**Table 2:** Descriptive statistics for fertility rate traits across Murciano-Granadina does.

Fertility rates	Minimum	Maximum	Mean	SD
Fertility by day of insemination	0.00	93.10	52.57	17.15
Fertility by buck batch and day of insemination	0.00	100.00	53.43	22.33
Fertility per day by semen type (fresh/chilled vs. frozen/thawed)	0.00	90.00	48.33	19.89

### Statistical analyses

#### Assessment of parametric assumptions

The choice of statistical methods was based on preliminary tests of parametric assumptions.

#### Normality

Distributional normality was evaluated using the Kolmogorov–Smirnov (K–S) test / Lilliefors correction test (suitable for samples over 5000), implemented through the *Test and Distribution Graphics* package in Stata v15.0 (StataCorp, College Station, TX, USA).

#### Homogeneity of variances

Refers to the assumption that the variance of a dependent variable is equal across all groups or levels of an independent variable. Tested in ANOVA or group comparisons. Variance homogeneity was assessed with Levene’s test via the *Explore* procedure in SPSS Statistics v25.0 (Armonk, NY, USA (IBM Corp, 2017).

#### Autocorrelation

First-order autocorrelation in model residuals was examined using the Durbin–Watson (DW) test (Durbin, 1970) in SPSS linear regression. The DW statistic (0–4 scale) is robust for *n* > 15 (Greenberg et al., 2020) and is particularly relevant for ordered temporal or spatial datasets (Chen, 2016), such as longitudinal testicular or semen measurements. A value near 2 indicates independence, values <2 indicate positive autocorrelation, and inconclusive outcomes arise when statistics fall between critical bounds (Gujarati & Porter, 2003).

#### Homoscedasticity

Specifically refers to constant variance of residuals across predicted values in a regression model. Checked via residual vs. fitted value plots in regression. Constant variance of residuals was checked using scatterplots of standardized predicted values against residuals, with residuals defined as the difference between observed and predicted scores. Heteroscedasticity was inferred when spread varied systematically, often narrowing at lower predicted values.

#### Dimensionality reduction of variate sets

Dimensionality reduction was applied to two datasets: fertility rates comprising fertility by day of insemination, fertility by buck batch and day of insemination, and fertility per day by semen type (fresh/chilled vs. frozen/thawed), and a second one comprising linear appraisal traits. The aim was to reduce data complexity while retaining the most informative variation. By transforming correlated variables into a smaller set of orthogonal components, this approach captured the main patterns in fertility and body conformation without loss of key biological information. It also facilitated detection of associations between reproductive and morphological traits, improved modeling efficiency, and enhanced interpretability by emphasizing principal sources of variation.

To ensure multicollinearity did not bias results, Variance Inflation Factor (VIF) and tolerance values were calculated for all predictors. Variables with VIF > 5 or tolerance < 0.1 were flagged for evaluation, but none required removal. Thus, all traits were retained in the final analyses, confirming that inter-predictor correlations were within acceptable limits for robust multivariate modeling.

#### Regularized generalized canonical correlation analysis (rCCA)

rCCA was conducted using XLSTAT 2014 and SPSS 25.0 syntax following González et al. (2008). Regularization was applied to reduce overfitting risks inherent in high-dimensional datasets with relatively small sample sizes, thereby increasing the robustness of detected linear associations (Parkhomenko et al., 2009).

Regularized canonical correlation analysis (rCCA) was selected because the primary objective of the study was to investigate the shared multivariate structure between two predefined and biologically distinct blocks of variables—linear appraisal traits (LAS-derived conformation measures) and fertility indicators—without imposing directional assumptions. The research question was not framed in predictive terms (i.e., predicting semen quality from morphology or vice versa), but rather in exploratory terms: to determine whether coherent patterns of covariance exist between overall body conformation and long-term reproductive dynamics.

Classical canonical correlation analysis (CCA) provides a natural framework for identifying maximally correlated linear combinations of two variable sets; however, the moderate dimensionality of each block relative to the effective sample size, combined with substantial internal collinearity among morphological descriptors, rendered standard CCA potentially unstable. rCCA extends the classical formulation by incorporating penalization, typically ridge-type regularization, which stabilizes coefficient estimation, reduces overfitting, and improves interpretability under multicollinearity (González et al., 2006; H.D. Vinod, 1976; Leurgans et al., 1993; Wells et al., 2024). This was particularly relevant given that linear appraisal traits are inherently correlated due to shared developmental and anatomical pathways.

Alternative multivariate approaches were considered but were less aligned with the analytical objectives. Partial Least Squares (PLS), although capable of handling multicollinearity, is fundamentally optimized for predictive variance in a dependent block, thereby introducing an implicit asymmetry between predictor and response sets (Tenenhaus et al., 2011). In contrast, the present study aimed to examine reciprocal structural association rather than prediction. Structural Equation Modeling (SEM) was also not appropriate, as it requires the specification of a priori causal pathways and latent constructs; the current design was exploratory and did not posit a directional mechanistic model linking specific conformational traits to fertility outcomes. Similarly, dimensionality-reduction techniques such as principal component analysis (PCA) operate within a single block and therefore do not directly quantify cross-block association. rCCA thus represents a methodologically coherent choice: it preserves the symmetric treatment of both trait domains, directly quantifies shared variance structure, and accommodates the modest but non-negligible multicollinearity characteristic of morpho-functional data in selected livestock populations. Within the biologically buffered and moderately dispersed fertility landscape of the Murciano-Granadina breeding nucleus, this framework allows subtle but structured multivariate relationships to be detected without overstating predictive strength or causal interpretation.

Accordingly, rCCA was employed as an exploratory tool to characterize coordinated variation between morphology and reproductive dynamics, with results interpreted in terms of association patterns rather than deterministic or predictive inference.

### Pearson’s product–moment correlations

After confirming acceptable levels of multicollinearity, Pearson’s correlations were calculated both within and between variable sets using XLSTAT 2014. Interpretation followed established guidelines (Gil-Lebrero et al., 2020; Profillidis & Botzoris, 2018).

#### Validity assessment

The reliability of rCCA results was strengthened by regularization, which minimizes overfitting in complex datasets. The significance of canonical correlations was evaluated with Pillai’s trace, a robust statistic that tolerates violations of normality, homoscedasticity, and independence (Olson, 1976). These tests were performed in STATA 16 (StataCorp, 2019).

#### Variance explanation

Eigenvalues, derived from the product of the model and inverse error matrices, represented squared canonical correlations. Larger eigenvalues indicated greater proportions of variance explained by the canonical variates.

#### Canonical correlations and redundancy

Canonical correlations ranged from –1 to 1 and were interpreted analogously to Pearson’s coefficients (Gil-Lebrero et al., 2020; Profillidis & Botzoris, 2018). Correlations ≥0.30 were considered meaningful, corresponding to roughly 10 % of variance explained. Redundancy coefficients were additionally computed to quantify the proportion of variance in one set accounted for by the other.

#### Roots

Roots corresponded to ordered eigenvalues, each testing the null hypothesis that its associated canonical correlation equaled zero. This procedure determined the number of dimensions necessary to adequately describe relationships between variable sets.

#### Wilks’ lambda and R²

Wilks’ lambda was calculated as the product of (1 − canonical correlation) across sets. Values approaching 0 indicated stronger associations, whereas values near 1 suggested modest or absent relationships (Tabachnick et al., 2007).

#### rCCA cross-validation

Model stability was assessed through ten-fold cross-validation in R 4.1.1 using the *CCA, RGCCA* and *mixOmics* packages (González et al., 2008; Lê Cao & Welham, 2021; R Core Team, 2020; Girka et al., 2023). Regularization parameters (λ1 and λ2) were optimized with the tune.rcc function to maximize cross-validation performance (González et al., 2009).

## Results

### Statistical analyses

#### Parametric assumption testing

Normality

Residuals of the modeled fertility parameters were assessed using the Kolmogorov–Smirnov test with Lilliefors correction. No significant deviations from normality were detected (*p* > 0.05), supporting the appropriateness of parametric methods.

#### Homogeneity of variances

Levene’s test confirmed that variances were homogeneous across groups (*p* > 0.05), indicating consistent variability in the residuals.

#### Autocorrelation

Durbin–Watson statistics for all models were close to 2, suggesting no evidence of first-order autocorrelation in the residuals.

#### Homoscedasticity

Scatterplots of residuals against predicted values showed constant variance across fitted values, indicating no heteroscedasticity.

#### Outlier analysis

The ROUT method (*Q* = 1 %) identified no extreme values that significantly distorted the dataset, as all candidates fell below the false discovery threshold (*p* < 0.01).

Collectively, these assessments indicate that the main parametric assumptions underlying multivariate analyses—namely normality of residuals, homogeneity of variances, absence of first-order autocorrelation, and homoscedasticity—were reasonably satisfied. Therefore, the application of parametric linear modeling approaches, including Pearson correlations and regularized canonical correlation analysis, was considered statistically appropriate for evaluating relationships between fertility and linear appraisal traits in Murciano-Granadina does.

#### Dimensionality reduction of variate sets

All variables in the linear appraisal set, including stature (height at withers), chest width, body depth, rump width, rump angle, angulosity, bone quality, anterior insertion, rear insertion height, median suspensory ligament, udder width, udder depth, nipple placement, nipple diameter, rear legs (rear and side views), and mobility, presented Variance Inflation Factor (VIF) values below 5, indicating the absence of problematic multicollinearity. A similar result was observed for the three fertility rate traits (fertility by day of insemination, fertility by buck batch and day of insemination, and fertility per day by semen type). These findings confirm that correlations among predictors remained within acceptable limits, ensuring the reliability of subsequent multivariate analyses.

### Regularized generalized canonical correlation analysis (rCCA)

#### Pearson’s product–moment correlations

The three fertility measures were positively correlated, indicating related but distinct patterns. Fertility by day of insemination showed a strong association with fertility by buck batch and day of insemination (*r* = 0.75), suggesting that day-to-day fertility trends are closely influenced by buck-specific effects. Fertility by day also correlated moderately with fertility per day by semen type (fresh/chilled vs. frozen/thawed; *r* = 0.67), indicating that overall daily fertility patterns are generally consistent across semen types. The correlation between fertility by buck batch and day and fertility per semen type was lower (*r* = 0.52), reflecting that both buck batch and semen type independently contribute to variability in fertility outcomes. Collectively, these results justify the use of multivariate approaches to summarize fertility variation across days, bucks, and semen types (Table 3).

**Table 3:** Pearson’s correlations between fertility rate traits. Color scale ranges from green (maximum positive value) to red (maximum negative value).

Correlation analysis among 17 linear appraisal traits revealed that skeletal measurements were generally interrelated. Stature (height at withers) showed moderate positive correlations with chest width (*r* = 0.55) and rump width (*r* = 0.54), indicating that taller does tended to have broader thoracic and pelvic structures. Chest width was also strongly associated with angulosity (*r* = 0.60). Bone quality was negatively correlated with stature (*r* = –0.41), chest width (*r* = –0.33), and rump width (*r* = –0.37), suggesting that larger, more angular does had flatter, neater bones. Mammary traits, such as udder depth and median suspensory ligament, were moderately associated (*r* = 0.42), whereas udder width and nipple traits showed low correlations with structural measurements (|r| ≤ 0.23), indicating relative independence. Rear legs – rear view and mobility were modestly positively correlated (*r* = 0.25), and rear legs – side view and mobility also showed a positive association (*r* = 0.22), reflecting the functional importance of correct leg structure. Overall, these patterns indicate that while body size and skeletal traits covary, mammary and appendage traits contribute independent variation, supporting multivariate approaches to summarize body conformation (Table 4).

**Table 4:** Pearson’s correlations between linear appraisal trait pairs In Murciano-Granadina does. Color scale ranges from green (maximum positive value) to red (maximum negative value).

Pearson correlation analysis between fertility traits and 17 linear appraisal traits revealed modest associations overall. The strongest negative correlations were observed for chest width (*r* = –0.14 with fertility by day of insemination) and rump width (*r* = –0.11), while bone quality showed the largest positive correlations (*r* = 0.04–0.06). Most other traits, including stature, body depth, udder measurements, rump angle, nipple traits, and mobility, displayed near-zero correlations (|r| ≤ 0.09), indicating minimal direct influence on fertility. These results suggest that reproductive performance in Murciano-Granadina does is largely independent of measured conformation traits, highlighting the need for multivariate approaches to detect any subtle associations between morphology and fertility (Table 5).

**Table 5:** Pearson’s correlations between fertility rate traits and linear appraisal trait pairs. Color scale ranges from green (maximum positive value) to red (maximum negative value).

#### Validity

**Fig. 5:**
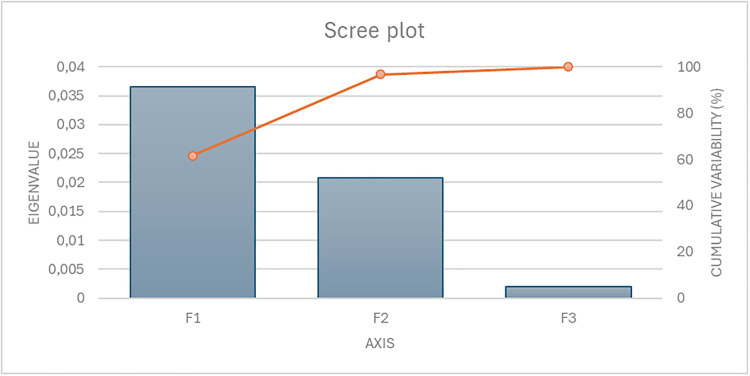
Scree plot of eigenvalues for canonical functions in Fertility by day of insemination (top), Fertility by buck batch and day of insemination (middle) and Fertility per day by semen type (fresh/chilled vs. frozen/thawed) (bottom) datasets, illustrating the proportion of variability explained by the three canonical functions.

The scree plot analysis of fertility and linear appraisal traits (Fig. 5) presents the eigenvalues and explained variance for three extracted factors (F1–F3). F1 has the largest eigenvalue (0.0366) and accounts for 61.62 % of the total variability, indicating it captures the majority of the information in the dataset. F2 contributes an additional 35.04 %, bringing the cumulative variance explained by the first two factors to 96.67 %, which suggests that most of the variability is effectively summarized by F1 and F2. F3 accounts for only 3.33 %, reflecting minimal additional information beyond the first two factors. Overall, this indicates that a two-factor solution is sufficient to capture the primary sources of variation in the data.

Table 6 summarizes the results of the multivariate canonical correlation analysis, including Wilks’ Lambda, F-tests, canonical correlations, and redundancy coefficients for three canonical functions (F1–F3). All three functions were statistically significant, with F1 (*F* = 17.37, *p* < 0.0001), F2 (*F* = 10.52, *p* < 0.0001), and F3 (*F* = 1.92, *p* = 0.0170), indicating that the canonical variates explain a significant portion of the shared variation between fertility traits and linear appraisal traits.

**Table 6:** Multivariate Tests of Significance and Canonical correlations and Redundancy coefficients.

Functions	F1	F2	F3
Wilks' Lambda	0.9415	0.9773	0.9980
F	17.3683	10.5190	1.9224
DF1	51	32	15
DF2	43,300.6198	29,090	14,546
Pr > F	< 0.0001	< 0.0001	0.0170
Canonical correlations	0.1913	0.1443	0.0445
Squared Canonical correlations	0.0366	0.0208	0.0020
Redundancy coefficients (Y1)	0.0072	0.0146	0.0002
Redundancy coefficients (Y2)	0.0053	0.0018	0.0001

The canonical correlations were relatively low (0.1913 for F1, 0.1443 for F2, and 0.0445 for F3), corresponding to squared canonical correlations of 0.0366, 0.0208, and 0.0020, respectively. This indicates that only a small proportion of variance in one set is linearly associated with the other. Redundancy coefficients were also low for both variable sets (Y1: 0.0072–0.0146; Y2: 0.0001–0.0053), suggesting that the canonical variates explain only a minor fraction of the total variance within each set.

Overall, the analysis reveals statistically significant but modest multivariate associations between fertility outcomes and linear appraisal traits, indicating that while body conformation contributes to reproductive variation, it explains only a limited proportion of the overall variability in fertility measures. Although the magnitude of the canonical correlations suggests restricted predictive capacity at the individual level, the detected associations reflect coherent patterns of structural–reproductive integration that may be biologically meaningful within a multifactorial and environmentally influenced fertility framework.

### Standardized coefficients and canonical discriminant functions

Standardized canonical coefficients were used to construct discriminant equations for each function, enabling the quantification of the relative contribution of individual traits to the observed multivariate associations. By examining these equations, we aimed to uncover biologically meaningful patterns linking structural and mammary conformation with reproductive performance, while accounting for the interdependence among traits.

Fertility Traits (F1–F3)

F1 (Fertility traits) = –1.1271 × Fertility by day + –0.2207 × Fertility by buck batch + 1.1234 × Fertility per semen type

Interpretation: F1 contrasts fertility by semen type (fresh/chilled vs. frozen/thawed) positively against daily fertility and buck-batch-specific fertility. Higher F1 scores reflect does with relatively higher fertility from semen type differences, highlighting the impact of semen preservation on reproductive performance.

F2 (Fertility traits) = 0.1716 × Fertility by day + 0.2004 × Fertility by buck batch + 0.7438 × Fertility per semen type

Interpretation: F2 emphasizes fertility from semen type more strongly, with modest contributions from daily and buck-specific fertility. This suggests that F2 captures secondary variation associated with semen type while less influenced by day-to-day or buck batch effects.

F3 (Fertility traits) = –1.3083 × Fertility by day + 1.4784 × Fertility by buck batch + 0.0158 × Fertility per semen type

Interpretation: F3 contrasts buck-batch-specific fertility against overall day-level fertility, with semen type having negligible influence. This function may reflect inherent differences among bucks or management-related variation in buck performance.

### Linear appraisal traits (F1–F3)

F1 (Linear traits) = 0.2043 × Stature + 0.4689 × Chest Width – 0.0223 × Body Depth – 0.0108 × Rump Width + 0.0174 × Rump Angle + 0.2736 × Angulosity + 0.1691 × Bone Quality – 0.1941 × Anterior Insertion + 0.1881 × Rear Insertion Height + 0.1636 × Median Suspensor Ligament – 0.1413 × Udder Width + 0.1171 × Udder Depth + 0.0009 × Nipple Placement + 0.0436 × Nipple Diameter + –0.1689 × Rear Legs Rear View + 0.4603 × Rear Legs Side View + 0.0399 × Mobility

Interpretation: F1 highlights structural size and body conformation, particularly chest width, side-leg structure, and angulosity, suggesting these traits co-vary and may subtly influence fertility variation captured by F1.

F2 (Linear traits) = 0.0472 × Stature – 0.5391 × Chest Width – 0.1253 × Body Depth – 0.2961 × Rump Width + 0.0582 × Rump Angle + 0.5487 × Angulosity + 0.1935 × Bone Quality – 0.0164 × Anterior Insertion – 0.0858 × Rear Insertion Height – 0.0617 × Median Suspensor Ligament – 0.3966 × Udder Width – 0.1172 × Udder Depth – 0.0514 × Nipple Placement + 0.0835 × Nipple Diameter + 0.0441 × Rear Legs Rear View + 0.4621 × Rear Legs Side View – 0.2305 × Mobility

Interpretation: F2 emphasizes angularity, chest width (negative), side-leg structure, and udder width, suggesting this function captures variation in skeletal angularity and udder conformation rather than overall size.

F3 (Linear traits) = 0.3104 × Stature + 0.7548 × Chest Width – 0.4860 × Body Depth – 0.4269 × Rump Width + 0.2031 × Rump Angle + 0.0763 × Angulosity – 0.1035 × Bone Quality – 0.1878 × Anterior Insertion + 0.5093 × Rear Insertion Height – 0.1183 × Median Suspensor Ligament – 0.2437 × Udder Width – 0.4626 × Udder Depth – 0.3346 × Nipple Placement + 0.2604 × Nipple Diameter + 0.2426 × Rear Legs Rear View – 0.1433 × Rear Legs Side View + 0.0113 × Mobility

Interpretation: F3 contrasts body width and depth traits (chest, rump, body depth) with rear udder and nipple morphology. It may capture a trade-off between overall body size and mammary traits, indicating that larger does may not necessarily have the most optimal udder conformation for fertility.

### Paired functions interpretation

Function 1 (Fertility F1 ↔ Linear F1)

• Fertility F1 emphasizes semen type (fresh/chilled vs. frozen/thawed) positively while contrasting it against day-level and buck-batch-level fertility (negative loadings).
• Linear F1 emphasizes structural conformation traits: chest width, rear leg side view, angulosity, and stature, indicating overall body frame and skeletal alignment.
• Interpretation: This function pair suggests that differences in fertility due to semen type are associated with body size and skeletal alignment. Does with stronger body frames and correct leg conformation may perform relatively better under frozen/thawed semen use, compensating for preservation-related fertility losses.

Function 2 (Fertility F2 ↔ Linear F2)

• Fertility F2 still emphasizes semen type, but with more modest contributions from day and buck effects, suggesting secondary variation.
• Linear F2 highlights angulosity and rear leg side view, while negatively weighting chest width and udder width. This contrasts angular skeletal features with compact udder morphology.
• Interpretation: This pair indicates that secondary fertility variation linked to semen type aligns with skeletal angularity and udder shape. It may reflect that does with angular frames and tighter udders show more resilience to semen type effects.

Function 3 (Fertility F3 ↔ Linear F3)

• Fertility F3 contrasts buck-batch fertility (positive) against day-level fertility (negative), with semen type having negligible influence.
• Linear F3 contrasts chest width and body size against udder depth, width, and teat traits, showing a trade-off between general size and mammary morphology.
• Interpretation: This pair captures buck-driven variation in fertility that is linked to body–udder trade-offs. Bucks with specific fertility profiles may perform differently depending on whether their daughters prioritize body capacity or optimal udder conformation.

### Canonical correlation analysis k-Fold cross-validation

**Fig. 6:**
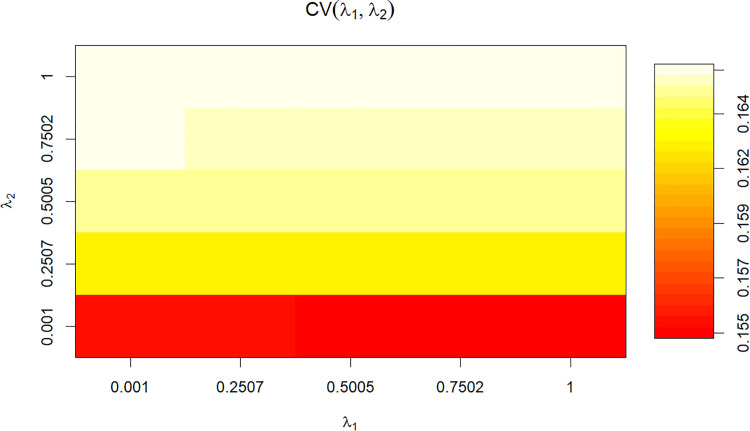
Optimal cross-validation scores for the values of the parameter of regularization (λ_1_ and λ_2_).

Fig. 6 heatmap shows the cross-validation (CV) coefficients obtained for combinations of λ₁ (x-axis) and λ₂ (y-axis). CV values range between 0.155 and 0.164. The lowest performance is observed in the red-shaded region, corresponding to λ₂ ≈ 0.001 across all λ₁ values, where CV values approach the minimum (≈ 0.155–0.157). Performance improves progressively as λ₂ increases, with yellow to white zones indicating higher CV coefficients. The best values (≈ 0.163–0.164) occur for λ₂ ≥ 0.5, regardless of λ₁, where the heatmap reaches its lightest tones. This indicates that CV performance is more strongly influenced by λ₂, with optimal results obtained at higher λ₂ values in combination with λ₁ ≈ 1.

## Discussion

Breeding programs in goats, such as those developed for the Murciano-Granadina breed, rely on a combination of strategies, including official milk yield recording, linear morphological appraisal, and artificial insemination (Delgado et al., 2017). Among these, artificial insemination (AI) is particularly valuable for optimizing genetic and productive efficiency, yet its success depends on understanding the factors that influence fertility. In this context, linear morphological appraisal emerges as a complementary tool to predict reproductive outcomes. Seventeen zoometric variables are assessed in females—seven more than in males—because several udder-related traits are essential in a dairy breed. These include stature (height at withers), chest width, body depth, rump width, rump angle, angulosity, bone quality, anterior and rear udder insertion, udder depth, udder width, median suspensory ligament, teat traits (placement and diameter), rear leg conformation (side and rear views), and mobility. This comprehensive set of descriptors enables the exploration of relationships between morphology and fertility.

The confirmation of parametric assumptions should be interpreted in light of the biological and organizational context of the Murciano-Granadina breeding program. This population does not represent an unmanaged or randomly structured genetic pool; rather, it operates under a long-standing and coordinated improvement scheme that integrates milk recording, artificial insemination, and linear morphological appraisal (Dirección General de Producciones y Mercados Agrarios, 2021; Mokhtari et al., 2024). Within such structured systems, animals presenting severe reproductive dysfunction or marked conformational imbalance are unlikely to remain within the breeding nucleus over time. As a result, fertility may be expected to behave as a relatively stabilized quantitative trait influenced predominantly by numerous small additive genetic and environmental effects, which is consistent with the moderate heritability estimates and polygenic architecture reported for reproductive traits in Spanish dairy goats (Mokhtari et al., 2024; Aguirre-Arroyo et al., 2024; Ziadi et al., 2021).

From this perspective, the approximately normal distribution of residuals and the absence of extreme outliers appear biologically plausible. Stabilizing selection, even when indirect, may reduce the frequency of highly subfertile phenotypes and contribute to greater functional homogeneity across herds. Although such statistical behavior does not in itself prove biological buffering, it aligns with expectations for a population subjected to sustained selection for functional efficiency.

Similarly, the observed homogeneity of variances, lack of detectable heteroscedasticity, and absence of temporal autocorrelation can be viewed in the context of standardized management practices and the controlled dissemination of genetics through artificial insemination (Argüello, 2011). In breeding systems focused exclusively on maximizing production, increases in variance or signs of reproductive instability are sometimes reported; however, the Murciano-Granadina program explicitly incorporates functional traits alongside productivity goals (Fernandez Alvarez et al., 2023). This broader selection objective may help maintain a relatively balanced dispersion of fertility outcomes.

The absence of temporal autocorrelation over the ten-year period further suggests that no progressive clustering of subfertile lines occurred within the dataset. This observation is compatible with previous evidence indicating that reproductive performance in dairy goats is shaped to a considerable extent by management conditions and insemination practices, in addition to underlying genetic effects (Castañeda-Bustos et al., 2014).

Taken together, these elements suggest that fertility variation in this selected dairy goat population can be interpreted as moderately dispersed and structurally stable within the limits of the evaluated data. Under such conditions, the application of parametric multivariate approaches appears statistically defensible. Importantly, the modest magnitude of the canonical associations should be interpreted within this biologically buffered framework, where subtle but coherent relationships are more plausible than large deterministic effects.

From a biological perspective, the selection of regularized canonical correlation analysis (rCCA) reflects the nature of the relationship under investigation: linear appraisal traits and fertility indicators represent two coordinated but distinct phenotypic domains shaped by shared developmental, endocrine, genetic, and management influences (Mrode, 2014). In the Murciano-Granadina goat, linear appraisal traits capture structural soundness, proportionality, and functional balance, whereas fertility indicators summarize realized reproductive success under practical breeding conditions (Fernández Álvarez et al., 2021). These domains interact biologically, but neither can be assumed to function as a strictly causal determinant of the other. Structural conformation may influence fertility through mechanisms such as body condition stability, locomotor soundness affecting estrus expression and mating efficiency, pelvic architecture influencing parturition ease, and overall robustness reflecting endocrine and metabolic resilience (Rauw et al., 1998; Kosgey, 2004). Conversely, reproductive history and the physiological demands of gestation and lactation may influence body composition and structural expression over time (GonzálezGarcía et al., 2014). The relationship is therefore better conceptualized as coordinated biological integration rather than directional prediction. rCCA preserves this biological symmetry by identifying pairs of latent linear combinations that maximize shared covariance between conformation and fertility blocks without imposing a dependent–independent hierarchy (González et al., 2008; H.D. Vinod, 1976). This is particularly appropriate in a population subjected to sustained stabilizing selection, where extreme maladaptive phenotypes are progressively removed and trait dispersion becomes functionally moderated (Hill & Kirkpatrick, 2010). Regularization is essential because multicollinearity is inherent in morphological datasets, where traits share developmental and anatomical underpinnings; without regularization, canonical coefficients become unstable and overly sensitive to sampling variation (Leurgans et al., 1993; Witten et al., 2009). Alternative multivariate approaches do not surpass rCCA in this context. Partial Least Squares (PLS) is optimized for predictive performance and introduces asymmetry by prioritizing variance explanation in a designated response block; biologically, this would imply that morphology primarily predicts fertility (or vice versa), overstating directional influence within a polygenic and management-dependent reproductive system (Abdi, 2010). Structural Equation Modeling (SEM) requires explicit causal pathways and latent constructs specified a priori; however, fertility in dairy goats is governed by numerous small additive genetic effects and substantial environmental modulation, and imposing directional causal structures without experimentally validated mechanisms could exaggerate biological certainty (Notter, 2012). Principal Component Analysis (PCA) and related single-block techniques summarize variance within domains but do not directly quantify cross-domain covariance, thereby failing to address the integrative biological question. Even classical CCA is less suitable due to ill-conditioned covariance matrices and multicollinearity, conditions under which regularized variants were specifically developed to improve stability and interpretability (González et al., 2008; Witten et al., 2009).

In addition, sparse or graphical variants of canonical correlation analysis were considered but not adopted. Although sparse CCA approaches (Parkhomenko et al., 2009; Witten et al., 2009) introduce penalization to perform variable selection and enhance interpretability in high-dimensional genomic contexts, the present study did not aim to identify a minimal subset of traits driving fertility variation. Instead, the objective was to characterize the coordinated latent structure between two biologically defined phenotypic domains in their entirety. Linear appraisal traits in dairy goats represent an integrated functional system shaped by shared developmental pathways, and excluding variables through sparsity constraints could artificially fragment this biological coherence. Therefore, ridge-type regularization was preferred over sparsity-inducing penalties to stabilize coefficient estimation while preserving the full morphofunctional architecture of the trait block. This approach aligns with the exploratory and integrative nature of the study, avoiding overinterpretation of individual trait contributions while retaining multivariate structure.

The biological coherence of the modest canonical correlations observed is consistent with empirical findings in goat populations, where conformation traits and reproductive performance often show low-to-moderate associations due to strong environmental influence and polygenic architecture (Fernández Álvarez et al., 2021; Okere et al., 2022). Canonical correlation studies in other caprine breeds similarly report structured but modest cross-domain relationships, reinforcing the expectation of modest deterministic coupling between morphology and reproductive outcomes (McLaren et al., 2016). In Murciano-Granadina goats specifically, multivariate analyses of linear appraisal traits have demonstrated strong internal correlations and genetic structuring, further supporting the need for regularization when linking these traits to other biological domains (Fernández Álvarez et al., 2021). rCCA therefore aligns both statistically and biologically with the integrated, polygenic, and management-mediated architecture of fertility in this population, enabling detection of coordinated but modest associations without imposing predictive asymmetry or unsupported causal inference.

The overall pattern of Pearson correlations between fertility indicators and the set of linear appraisal traits suggests that reproductive performance in Murciano-Granadina goats is only modestly associated with conformational characteristics when evaluated independently. Correlation coefficients ranged approximately between −0.14 and 0.09 across the different fertility definitions, indicating subtle but directionally structured associations rather than random variation. Certain traits—including Chest Width, Rump Width, and Stature—showed consistent negative tendencies across fertility indicators, whereas others, such as Bone Quality and Rear Legs (side view), displayed neutral to slightly positive associations depending on the fertility definition considered. These magnitudes are consistent with expectations for complex reproductive traits and reflect biologically coherent patterns (Mocé et al., 2022).

Importantly, the interpretation of these results must be contextualized within the genetic architecture of both conformation and reproductive traits. According to Fernández Álvarez et al. (2021), heritability estimates for linear appraisal traits in Murciano-Granadina goats range from low to moderate values (0.09–0.43), indicating meaningful additive genetic control and response to sustained selection within the breeding program. In contrast, reproductive traits in this breed exhibit extremely low heritability estimates, ranging from 0.02 to 0.07 for litter size and from 0.02 to 0.03 for total litter weight (Mokhtari et al., 2024). These values demonstrate that reproductive performance is predominantly influenced by environmental, management, and physiological factors rather than by additive genetic variance.

The very low heritability of fertility traits provides a clear explanation for the modest phenotypic correlations observed in the present study. When a trait exhibits minimal genetic variability, the majority of its phenotypic variation arises from non-genetic sources. Consequently, even biologically relevant structural influences are expected to generate only small observable correlations at the phenotypic level. The correlation magnitudes detected (|r| up to approximately 0.14) are therefore consistent with the expected effect sizes when relating moderately heritable morphological traits to highly environmentally driven reproductive outcomes.

From a physiological perspective, reproductive performance emerges from coordinated genetic, endocrine, metabolic, and environmental interactions that extend beyond static morphological descriptors. Structural conformation may reflect long-term developmental and functional status; however, fertility outcomes depend on dynamic processes such as follicular activity, uterine environment, metabolic balance, and hormonal regulation. Moreover, pregnancy status, lactational stage, and seasonal influences can induce subtle but biologically meaningful structural variation (GonzálezGarcía et al., 2014). When conformation scoring does not temporally coincide with critical reproductive windows—such as insemination or early gestation—associations may appear attenuated in cross-sectional analyses, even when underlying biological links exist.

Methodologically, linear appraisal scoring in this study was conducted by a single trained evaluator, ensuring consistency and minimizing inter-observer variability. This strengthens the internal reliability of the phenotypic assessments. Nevertheless, linear scoring systems operate within bounded categorical scales, which may constrain phenotypic variance and limit detectable effect sizes when relationships are subtle. Thus, the modest correlations observed may partially reflect measurement scale properties rather than absence of biological relevance.

Additionally, fertility outcomes are strongly influenced by environmental and management factors—including nutrition, seasonality, and male effect—which can interact with structural characteristics in complex and context-dependent ways (Mocé et al., 2022). Furthermore, seasonal reproductive variability inherent to small ruminants may attenuate linear associations, as endocrine cyclicity, estrus expression, and ovulatory dynamics fluctuate across photoperiodic transitions. Within such biologically dynamic systems, morphological influences may operate conditionally or within optimal physiological ranges rather than along strictly linear gradients. Consequently, simple bivariate correlations may underestimate coordinated structural contributions to reproductive performance.

Taken together, the coexistence of moderate heritability for conformation traits and extremely low heritability for reproductive traits provides a coherent genetic framework for interpreting the observed results. Structural traits are genetically responsive, whereas fertility traits are predominantly environmentally determined. Therefore, the modest phenotypic associations reported should not be interpreted as evidence of independence, but rather as the expected outcome of relating traits with contrasting genetic architectures. Morphological contributions to fertility are likely indirect and expressed through integrated biological pathways rather than through dominant one-to-one effects. These findings reinforce the importance of applying integrative multivariate approaches to better characterize the complex structural–reproductive interactions underlying fertility performance. Given the low heritability of reproductive traits and the modest magnitude of univariate correlations, fertility is unlikely to be adequately explained by isolated structural descriptors. Instead, coordinated patterns of morphology and reproductive responses should be considered to capture the biological complexity of the system.

More specifically, the linear appraisal trait functions (L-F1 to l-F3) provided complementary insight into structural and mammary correlates of fertility variation. l-F1 grouped skeletal size and alignment traits—particularly chest width, rear leg side view, and angulosity—highlighting the role of body frame and locomotor soundness in fertility resilience. l-F2 emphasized skeletal angularity and rear leg alignment, contrasting these traits with udder and chest width, suggesting that more angular, lighter-framed animals with compact udders may contribute differently to fertility variation. l-F3 contrasted body capacity (chest width and body depth) against mammary morphology (udder width, depth, and teat traits), reflecting a potential trade-off between general body size and udder conformation.

Fertility traits were similarly summarized into three complementary functions (F1–F3). F1 highlighted the influence of semen type (fresh/chilled versus frozen/thawed) while contrasting it with fertility by day and by buck batch. Higher F1 scores reflected does more resilient to fertility reductions associated with frozen/thawed semen. F2 continued to emphasize semen type, but to a lesser extent, capturing subtler variability in reproductive response. F3 shifted focus toward contrasts between fertility at the buck-batch level and day-to-day fertility, largely independent of semen type, indicating that male-specific and management-related factors also contribute substantially to fertility variation.

Although the magnitude of the canonical associations is modest, their interpretation must be contextualized within the biological architecture of fertility in this population. Reproductive performance in Murciano-Granadina goats exhibits very low heritability and is strongly modulated by environmental and management factors, as demonstrated in recent fertility prediction studies for this breed (Mocé et al., 2022). Under such conditions, large deterministic associations between static morphological descriptors and realized fertility would not be biologically expected. Across the canonical dimensions, 5.94 % of the shared multivariate covariance between fertility and morphology was captured, indicating weak but structured cross-domain integration. However, redundancy indices indicate that morphology accounts for up to approximately 1.5 % of total fertility variance, confirming that the direct explanatory contribution remains limited.

The scree plot indicates that Factor 1 (F1) accounts for 61.62 % of the total variability, representing the dominant axis of shared variation between fertility and linear appraisal traits. From a biological standpoint, this primary dimension likely reflects overall structural robustness and functional anatomical balance. In dairy goats, skeletal capacity, thoracic development, pelvic width, and limb alignment are not merely aesthetic descriptors but indicators of developmental stability, metabolic capacity, and locomotor soundness (Fernandez Alvarez et al., 2023; Rauw et al., 1998). Animals with balanced body frames and correct hind limb conformation are generally more capable of sustaining high milk production while maintaining reproductive efficiency, as they experience fewer locomotor constraints, better nutrient partitioning, and reduced physiological stress (Nascimento et al., 2021). Therefore, F1 can be interpreted as a global morpho-functional axis integrating structural soundness with reproductive resilience rather than as a simple statistical abstraction.

Factor 2 (F2), which explains an additional 35.04 % of variance (bringing cumulative explained variance to 96.67 %), appears to capture a secondary but biologically meaningful dimension of conformational variation. While F1 reflects overall robustness, F2 likely represents contrasts in dairy specialization—particularly angularity, body depth, and mammary configuration. In highly selected dairy populations, increased angularity and body refinement are often associated with elevated milk yield potential (Mellado et al., 2008). However, extreme dairy conformation may predispose animals to negative energy balance, which can impair ovarian function, delay estrus resumption, and reduce conception rates (González-García et al., 2015). Thus, F2 may represent a physiological trade-off axis between productive specialization and reproductive stability. This dimension is consistent with established biological principles in dairy species, where metabolic prioritization toward lactation can influence fertility outcomes (Rauw et al., 1998).

Importantly, Factor 3 (F3) explains only 3.33 % of total variability and does not appear to represent a distinct biological process beyond minor residual contrasts between body size and mammary traits. Given its minimal contribution to overall variance, F3 is unlikely to capture a coherent physiological pathway and therefore does not warrant extensive biological interpretation.

Taken together, the two-factor solution reflects the integrative nature of fertility in managed dairy goats. Reproductive performance does not depend on isolated morphological traits but rather on coordinated structural balance, metabolic stability, and mammary functionality. The dominance of F1 and F2 suggests that morpho–reproductive relationships in this population operate along two principal biological axes: (1) structural robustness and locomotor-functional integrity, and (2) dairy specialization versus reproductive balance. These findings align with the polygenic and multifactorial architecture of fertility, where subtle but coordinated anatomical characteristics may influence reproductive outcomes within environmentally modulated systems (Mocé et al., 2022).

When fertility and linear functions were paired, clearer biological interpretations emerged. Pairing F1 (fertility) with l-F1 suggested that fertility variation due to semen type is associated with skeletal size and structural soundness, implying that larger, well-aligned does may better tolerate reduced fertilization efficiency from frozen/thawed semen, potentially due to improved uterine environment or reproductive robustness. Pairing F2 with l-F2 reflected secondary fertility variation from semen type aligned with skeletal angularity and compact udder morphology, suggesting enhanced adaptability under varying semen preservation conditions. Finally, pairing F3 with l-F3 captured buck-driven fertility differences linked to body–udder trade-offs. This indicates that buck-specific fertility is expressed differently in daughters depending on whether body size or udder morphology is prioritized, highlighting a genetic and management interaction shaping reproductive outcomes.

Fertility was evaluated from three complementary perspectives: fertility by day of insemination, accounting for shared environmental conditions such as weather (Abecia et al., 2016; Arrebola et al., 2013; Arrébola et al., 2016); fertility by buck batch on the insemination day, reflecting ejaculate- or buck-specific performance across herds (Karagiannidis et al., 2000); and fertility by semen type, contrasting reproductive success with fresh/chilled versus frozen/thawed semen (Borges-Silva et al., 2016). While fresh/chilled semen predominates in practice, frozen/thawed semen is strategically important for long-distance dissemination of genetics despite its lower fertility. A consistent association was observed between fertility per day of insemination and fertility per buck batch with bone quality, a trait valued in dairy animals due to its flat, neat bones linked to high milk potential (Tadesse et al., 2022). Physiologically, the skeletal system enables efficient nutrient utilization and acts as a calcium reservoir for milk synthesis (Liesegang et al., 2000). Animals capable of efficient mineral mobilization sustain high milk yields while maintaining energy balance, supporting fertility (Castañeda-Bustos et al., 2017a). Similarly, rear udder insertion height showed a positive relationship with fertility per day and per buck batch. Proper attachment indicates functional mammary conformation, reducing udder injuries and mastitis (Akter et al., 2020; Miles et al., 2021; Singh et al., 2014), which can negatively impact fertility (Hudson et al., 2012). For fertility by semen type (fresh/chilled vs. frozen/thawed), rear leg side view (VLPT) showed a positive relationship. Correct hind leg structure is critical as these limbs bear more weight and influence mobility (Rodríguez et al., 2009). Goats with parallel, open, strong hind limbs access resources more efficiently, experience fewer injuries, and maintain udder integrity (Adams et al., 1979; Wall et al., 2005), indirectly enhancing reproductive efficiency. Other skeletal measures, such as greater stature and body depth, tended to oppose fertility, consistent with reports that larger, dominant goats may prioritize growth and milk production over reproduction (Zuñiga-Garcia et al., 2020; Walsh et al., 2011).

Morphological traits must be interpreted carefully, as higher linear scores do not always reflect better conformation. For example, udder depth is ideal when it reaches the hock level; excessively deep udders, despite high scores, increase mastitis risk and hinder milking (Bharti et al., 2015; Bhutto et al., 2010). Accordingly, deeper udders were negatively associated with fertility in this study. Mammary traits thus reflect both productivity and functional health, highlighting their dual importance in breeding programs (Chegini et al., 2019). Overall, associations between fertility and morphological traits were statistically significant but generally modest. Evidence suggests that selection for dairy morphology and high milk output in Murciano-Granadina goats may have inadvertently compromised reproductive efficiency. Bone quality, mammary attachment, and hind limb conformation emerged as particularly relevant traits, simultaneously supporting production, fertility, and longevity. Conversely, traits associated with extreme dairy specialization—such as high angularity, large body size, and deep udders—appeared detrimental to fertility, likely through negative energy balance and health-related pathways.

These results underscore that fertility variation in does is not solely determined by semen preservation or buck effects but is modulated by structural and mammary conformation. Skeletal robustness and udder morphology appear to buffer or amplify fertility outcomes under different reproductive technologies. In practice, smaller, functionally conformed does—with sound udders, strong hind limbs, and good bone quality—tend to optimize resource allocation, sustain fertility, and extend productive life. Integrating both reproductive management strategies and linear appraisal in breeding programs is therefore crucial. Given fertility’s multifactorial nature, further studies incorporating physiological, genetic, and management variables are needed to fully disentangle these complex relationships.

## Conclusions

This study demonstrates that combining artificial insemination with linear morphological appraisal enhances fertility management in Murciano-Granadina goats. Although overall canonical correlations between morphology and fertility were modest, specific traits emerged as particularly relevant. Skeletal robustness, correct hind leg conformation, and bone quality consistently supported fertility resilience, while functional udder attachment reduced health risks and improved reproductive outcomes. These findings confirm that structural soundness and mammary integrity are crucial pillars of reproductive success. Angular frames and more compact udders showed subtle but meaningful links with fertility variation, suggesting that lighter-framed, functionally balanced does may adapt better to the reproductive challenges posed by different semen types. Conversely, excessive stature, large body size, and overly deep udders were detrimental to fertility, likely through negative energy balance, mastitis risk, and reduced reproductive health. Overall, the results highlight the importance of balanced selection criteria in breeding programs. By prioritizing bone quality, udder support, and hind limb soundness, while avoiding extreme specialization for size or udder depth, breeders can optimize both fertility and longevity without compromising dairy productivity. The integration of reproductive technologies such as AI with targeted morphological appraisal provides a powerful framework for sustaining fertility, enhancing herd efficiency, and ensuring the continued success of the Murciano-Granadina breed.

## Funding

Funding was not received for the development of the present study. The present research was carried out during the covering period of a Ramón y Cajal Post-Doctoral Contract with the reference MCIN/AEI/10.13039/501100011033 and the European Union “NextGenerationEU”/PRTR.

## Ethical statement

The study followed the premises described in the Declaration of Helsinki. The Spanish Ministry of Economy and Competitivity through the Royal Decree-Law 53/2013 and its credited entity the Ethics Committee of Animal Experimentation from the University of Córdoba permitted the application of the protocols present in this study as cited in the fifth section of its second article, as the animals assessed were used for credited zootechnical use. This national Decree follows the European Union Directive 2010/63/UE, from the 22nd of September of 2010. Furthermore, the present study works with records rather than live animals directly, and these records were obtained after minimal handling, hence no special permission was compulsory.

## Disclosure statement

No potential conflict of interest was reported by the author(s).

## Data availability

Data will be made available from the corresponding author F.J.N.G. upon reasonable request.

## CRediT authorship contribution statement

**María Pía Peláez Caro:** Writing – original draft, Software, Resources, Methodology, Investigation, Formal analysis, Data curation. **Ander Arando Arbulu:** Writing – review & editing, Visualization, Validation, Supervision, Software, Methodology, Investigation, Formal analysis, Data curation. **José Manuel León Jurado:** Writing – review & editing, Visualization, Validation, Supervision, Software, Project administration, Methodology, Investigation, Formal analysis, Data curation. **Juan Vicente Delgado Bermejo:** Writing – review & editing, Visualization, Validation, Supervision, Resources, Project administration, Methodology, Investigation, Funding acquisition, Conceptualization. **Javier Fernández Álvarez:** Writing – review & editing, Visualization, Validation, Supervision, Software, Resources, Project administration, Investigation, Funding acquisition, Conceptualization. **Francisco Javier Navas González:** Writing – review & editing, Writing – original draft, Visualization, Validation, Supervision, Software, Methodology, Investigation, Formal analysis, Data curation, Conceptualization.

## Declaration of competing interest

I have The authors declare that they have no known competing financial interests or personal relationships that could have appeared to influence the work reported in this paper.

## Appendix Supplementary materials

Image, application 1
